# Higher Lifetime Stress and Symptom Burden Contribute to the Occurrence of Shortness of Breath

**DOI:** 10.1016/j.soncn.2023.151471

**Published:** 2023-07-25

**Authors:** Joosun Shin, Kord Kober, Patsy Yates, Melisa L. Wong, Bruce A. Cooper, Steven M. Paul, Marilyn Hammer, Yvette Conley, Jon D. Levine, Fay Wright, Christine Miaskowski

**Affiliations:** a Department of Physiological Nursing, School of Nursing, University of California, San Francisco, California; b Cancer & Palliative Outcomes Centre, Centre for Health Transformation, Faculty of Health, Queensland University of Technology, Brisbane, Queensland, Australia; c Department of Medicine, School of Medicine, University of California, San Francisco, California; d The Phyllis F. Cantor Center for Research in Nursing and Patient Care Services, Dana Farber Cancer Institute, Boston, Massachusetts; e Department of Health Promotion and Development, School of Nursing, University of Pittsburgh, Pittsburgh, Pennsylvania; f Rory Meyers College of Nursing, New York University, New York, New York; g Departments of Physiological Nursing and Anesthesia, School of Nursing and School of Medicine, University of California, San Francisco, California

**Keywords:** Adverse childhood experiences, Cancer, Depression, Post-traumatic stress disorder, Resilience, Shortness of breath, Stress

## Abstract

**Objectives:**

Among four classes of patients with distinct shortness of breath profiles, evaluate for differences in levels of global, cancer-specific, and cumulative life stress, as well as resilience; evaluate for differences in the occurrence rates for various stressful life events, and evaluate for differences in the severity of common co-occurring symptoms.

**Data Sources:**

Outpatients (N = 1338) completed questionnaires six times over two cycles of chemotherapy. The occurrence of shortness of breath was assessed using the Memorial Symptom Assessment Scale. Latent class analysis was used to identify subgroups of patients with distinct shortness of breath profiles. Differences among the classes were evaluated using parametric and nonparametric tests.

**Conclusion:**

Shortness of breath classes were labeled based on their distinct occurrence trajectories: None (70.5%), Decreasing (8.2%), Increasing (7.8%), and High (13.5%). Compared to None class, Decreasing and High classes had higher global and cancer-specific stress scores. The High class reported higher occurrence rates for several adverse childhood experiences. Compared to None class, Decreasing and High classes had higher depression, anxiety, and morning fatigue scores and lower morning energy and cognitive function scores.

**Implications for Nursing Practice:**

Given the additive or synergistic relationships between stress, co-occurring symptoms, and shortness of breath, multimodal interventions that include stress management, exercise training, and/or symptom management may decrease shortness of breath in oncology patients.

## INTRODUCTION

The 10% to 70% occurrence rate for shortness of breath in oncology patients provides evidence of the large amount of inter-individual variability in this symptom.^1^ However, limited information is available on factors that contribute to this variability.^2^ Therefore, effective management of shortness of breath is extremely challenging.^3^ Unrelieved shortness of breath is associated with decreases in functional status and quality of life,^4^ as well as overall survival.^5^

Given the paucity of research on risk factors for shortness of breath in oncology patients, our team developed the Multifactorial Model of Dyspnea in Patients with Cancer.^6^ The six factors included in this model are based on a synthesis of 19 studies on the occurrence^7–9^ and severity^4,10–24^ of dyspnea in oncology patients. The specific factors in this model include: person (ie, age, gender, socioeconomic status), clinical (ie, smoking, respiratory disease, heart disease), and cancer-related (eg, lung cancer, cancer treatments, anemia) factors, as well as respiratory muscle weakness, co-occurring symptoms (eg, anxiety, depression, fatigue), and stress. Given that the majority of the studies that provided the foundation for our model evaluated patients with lung cancer, additional research is warranted on risk factors for shortness of breath in patients with heterogeneous types of cancer undergoing chemotherapy.

Latent class analysis (LCA) is a person-centered analytic approach that can be used to identify modifiable and nonmodifiable risk factors associated with subgroups (ie, latent classes) of patients with distinct symptom profiles.^25^ Using this analytic approach, we evaluated for subgroups of patients with distinct shortness of breath profiles.^26^ In brief, in a sample of 1338 patients undergoing chemotherapy, 70.5% did not report shortness of breath. Of the remaining 395 patients, three distinct shortness of breath profiles were identified (ie, Decreasing (8.2%), Increasing (7.8%), and High (13.5%)). Consistent with our conceptual model, risk factors for membership in the High class included: older age, being unemployed, having a history of smoking, reporting a diagnosis of lung disease, having lung cancer, and having received a higher number of cancer treatments.

While this study provides new information on various demographic and clinical characteristics associated with the occurrence of shortness of breath in patients with heterogeneous types of cancer receiving chemotherapy,^26^ differences in the severity of other common co-occurring symptoms and various types of stress and resilience among the classes were not evaluated. As noted in our recent systematic review,^2^ studies on the associations between dyspnea and co-occurring symptoms in oncology patients are extremely limited. In addition, despite the growing body of evidence on the role of stress in oncology patients’ symptom experiences,^27–29^ no studies were identified that evaluated for associations between shortness of breath and stress.^2^

Existing evidence suggests that the hypothalamus is involved in the regulation of respiration under stress.^30,31^ In one cross-sectional study of patients with chronic shortness of breath,^32^ higher levels of perceived stress were associated with more severe shortness of breath. In addition, moderate to severe shortness of breath in these patients was associated with flatter diurnal cortisol slopes.^32^ While these findings suggest that chronic moderate to severe shortness of breath causes hypothalamic-pituitary-adrenal (HPA) axis dysregulation, additional studies are warranted because only 26.5% of these patients had cancer.

A cancer diagnosis and subsequent treatment(s) are significant stressful life events (SLEs).^33^ As noted in one review,^34^ 7.3% to 13.8% of oncology patients meet the criteria for post-traumatic stress disorder (PTSD) and an additional 10% to 20% meet the criteria for subsyndromal PTSD. These data suggest that cancer-specific SLEs have additive effects on the HPA axis that may affect the perception of dyspnea. While no studies examined the association between shortness of breath and cancer-specific stress, in a study of patients with chronic obstructive pulmonary disease (COPD),^35^ positive correlations were found between the severity of shortness of breath and measures of perceived stress and fear of COVID-19.

On the other hand, resilience corresponds to an individual’s protective attributes that promote successful adaptation to stressors.^36^ In one study of patients with non–small cell lung cancer,^37^ individuals with a lower functional status were more likely to have severe dyspnea and lower resilience. In another study of patients with COPD,^38^ higher levels of dyspnea were associated with lower levels of resilience. Equally important, a growing body of evidence suggests that early life stress plays a crucial role in shaping an individual’s adaptive and maladaptive responses to a variety of stressors in adulthood.^31^ Therefore, additional studies are warranted that evaluate for associations between dyspnea and various types of stress (ie, global, cancer-specific, cumulative life stress) and resilience in the same sample of oncology patients.

Of note, common symptoms (eg, anxiety, sleep disturbance) experienced by oncology patients may co-occur with shortness of breath. For example, higher levels of anxiety and depression were associated with more severe dyspnea in oncology patients.^9,11,15,24,39–46^ However, among these studies, only four^9,15,24,39^ included patients with heterogeneous types of cancer, none evaluated both trait and state anxiety, and the majority of the analyses were limited to correlation coefficients.^15,39,41,43–46^

While less well studied, fatigue is another symptom that demonstrates a positive relationship with dyspnea.^15,16,39,42,44^ In addition, in four studies,^11,21,24,42^ positive associations were found between dyspnea and pain. Of note, in two studies of patients with advanced cancer,^15,39^ dyspnea was positively correlated with sleep disturbance. Finally, cognitive impairment is a common symptom in patients undergoing chemotherapy.^47^ However, while a positive association was reported between dyspnea and mild cognitive impairment in patients with COPD, asthma, or heart failure,^48^ no studies have evaluated this association in oncology patients.

Given the paucity of research on the associations between the occurrence of dyspnea and stress and other common symptoms in oncology patients, the purpose of this study was to extend our previous findings that identified subgroups of patients with distinct shortness of breath profiles^26^ evaluate for differences in levels of global, cancer-specific, and cumulative life stress, as well as resilience among the four shortness of breath classes. In addition, differences among the four classes in the occurrence rates for various SLEs and the severity of common co-occurring symptoms were evaluated.

## METHODS

### Patients and Settings

This study is part of a larger, longitudinal study of the symptom experience of oncology outpatients receiving chemotherapy.^49^ Eligible patients were ≥18 years of age; had a diagnosis of breast, gastrointestinal, gynecological, or lung cancer; had received chemotherapy within the preceding 4 weeks; were scheduled to receive at least two additional cycles of chemotherapy; were able to read, write, and understand English; and gave written informed consent. Patients were recruited from two Comprehensive Cancer Centers, one Veterans Affairs hospital, and four community-based oncology programs during their first or second cycle of chemotherapy. The major reason for refusal was being overwhelmed with their cancer treatment.

### Study Procedures

Study was approved by the Committee on Human Research at the University of California, San Francisco and by the institutional review board at each of the study sites. Written informed consent was obtained from all patients. Patients completed the shortness of breath measure, a total of six times over two cycles of chemotherapy. Stress and symptom measures were completed at enrollment (ie, prior to the second or third cycle of chemotherapy). Medical records were reviewed for disease and treatment information.

### Instruments

#### Demographic and Clinical Measures

Patients completed a demographic questionnaire, Karnofsky Performance Status (KPS) scale,^50^ Self-Administered Comorbidity Questionnaire (SCQ),^51^ Alcohol Use Disorders Identification Test (AUDIT),^52^ and smoking history questionnaire. Level of exercise was assessed using an investigator developed questionnaire. Using the recommendation for physical activity from the Office of Disease Prevention and Health Promotion’s Healthy People 2020 report,^53^ patients’ responses were categorized into one of three exercise groups (ie, no exercise, <150 minutes per week, ≥150minutes per week).^54^ Medical records were reviewed for disease and treatment information.

#### Measure of Shortness of Breath

The shortness of breath item from the Memorial Symptom Assessment Scale (MSAS) was used to assess for the occurrence of shortness of breath (ie, yes or no) at each of the six assessments. Validity and reliability of the MSAS are well established.^55^

#### Stress and Resilience Measures

The 14-item Perceived Stress Scale (PSS) was used as a measure of global perceived stress according to the degree that life circumstances are appraised as stressful over the course of the previous week.^56^ Each item was rated on a 0-to-4 Likert scale (ie, 0 = never, 1 = almost never, 2 = sometimes, 3 = fairly often, 4 = very often). Total PSS scores can range from 0 to 56. Its Cronbach *α* was 0.89.

The 22-item Impact of Event Scale-Revised (IES-R) was used to measure cancer-related distress.^57,58^ Patients rated each item based on how distressing each potential difficulty was for them during the past week “with respect to their cancer and its treatment.” Each item was rated on a 0 (not at all) to 4 (extremely) Likert scale. Three sub-scales evaluate levels of intrusion, avoidance, and hyperarousal perceived by the patient. The total score can range from 0 to 88. Sum scores of ≥24 indicate clinically meaningful post-traumatic symptomatology, and scores of ≥33 indicate probable PTSD.^59^ Cronbach *α* for the IES-R total score was 0.92.

The 30-item Life Stressor Checklist-Revised (LSC-R) is an index of lifetime trauma exposure (eg, being mugged, sexual assault).^60^ The total LSC–R score is obtained by summing the total number of events endorsed (range of 0 to 30). If the patient endorsed an event, the patient was asked to indicate how much that stressor affected their life in the past year, from 1 (not at all) to 5 (extremely). These responses were summed to yield a total “affected” sum score. In addition, a PTSD sum score was created based on the number of positively endorsed items (out of 21) that reflect the DSM-IV PTSD Criteria A for having experienced a traumatic event.

The 10-item Connor-Davidson Resilience Scale (CDRS) evaluates a patient’s personal ability to handle adversity (eg, “I am able to adapt when changes occur”).^61,62^ Items are scored on a 5-point Likert scale (“not true at all” to “true nearly all of the time”). Total scores range from 0 to 40, with higher scores indicative of higher self-perceived resilience. The normative adult mean score in the United States is 31.8 (standard deviation [SD], 5.4),^62,63^ with an estimated minimal clinically important difference of 2.7.^64^ Its Cronbach *α* was 0.90.

#### Symptom Measures

The 20-item Center for Epidemiological Studies-Depression scale (CES-D) evaluates the major symptoms in the clinical syndrome of depression. A total score can range from 0 to 60, with scores of ≥16 indicating the need for individuals to seek clinical evaluation for major depression. The CES-D has well established validity and reliability.^65–67^ In this study, its Cronbach *α* was 0.89.

The 20-items on the Spielberger State-Trait Anxiety Inventory (STAI-S and STAI-T) were rated from 1 to 4.^68^ The STAI-S measures a person’s temporary anxiety response to a specific situation or how anxious or tense a person is “right now” in a specific situation. The STAI-T measures a person’s predisposition to anxiety as part of one’s personality. Cut-off scores of ≥31.8 and ≥32.2 indicate a high level of trait and state anxiety, respectively. Cronbach *α* values for the STAI-T and STAI-S were 0.92 and 0.96, respectively.

The 18-item Lee Fatigue Scale (LFS) was designed to assess physical fatigue and energy.^69^ Each item was rated on a 0 to 10 numeric rating scale (NRS). Total fatigue and energy scores were calculated as the mean of the 13 fatigue items and the 5 energy items, respectively. Higher scores indicate greater fatigue severity and higher levels of energy. Using separate LFS questionnaires, patients were asked to rate each item based on how they felt within 30 minutes of awakening (ie, morning fatigue, morning energy) and prior to going to bed (ie, evening fatigue, evening energy). The LFS has established cut-off scores for clinically meaningful levels of fatigue (ie, ≥3.2 for morning fatigue, ≥5.6 for evening fatigue) and energy (ie, ≤6.2 for morning energy, ≤3.5 for evening energy).^70^ Cronbach *α* values were 0.96 for morning and 0.93 for evening fatigue and 0.95 for morning and 0.93 for evening energy.

The 21-item General Sleep Disturbance Scale (GSDS) was designed to assess the quality of sleep in the past week.^71^ Each item was rated on a 0 (never) to 7 (everyday) NRS. The GSDS total score is the sum of the 21 items that can range from 0 (no disturbance) to 147 (extreme sleep disturbance). Higher total scores indicate higher levels of sleep disturbance. A GSDS total score of ≥43 indicates a significant level of sleep disturbance.^70^ Cronbach *α* for GSDS score was 0.83.

The 16-item Attentional Function Index (AFI) assesses an individual’s perceived effectiveness in performing daily activities that are supported by attention and working memory.^72^ A higher total mean score on a 0 to 10 NRS indicates better cognitive function.^72^ Total scores are grouped into categories of attentional function (ie, <5, low function; 5.0–7.5, moderate function; >7.5, high function).^73^ Cronbach *α* for the total AFI score was 0.93.

The occurrence of pain was evaluated using the Brief Pain Inventory.^74^ Patients who responded yes to the question about having pain were asked to indicate if their pain was or was not related to their cancer treatment. Patients were categorized into one of four groups (ie, no pain, only noncancer pain, only cancer pain, both cancer and noncancer pain). Patients rated the intensity of their worst pain using 0 (none) to 10 (excruciating) NRS. In addition, they provided information on pain’s level of interference with function.

### Data Analysis

Descriptive statistics and frequency distributions were generated for sample characteristics at enrollment using the Statistical Package for the Social Sciences (SPSS) version 28 (IBM Corporation, Armonk, NY). As was done previously,^75^ unconditional LCA was used to identify shortness of breath profiles that characterized unobserved subgroups of patients (ie, latent classes) over the six assessments. Before performing the LCA, patients who reported the occurrence of shortness of breath for ≤1 of the six assessments were identified and labeled as the “None” class (n = 943, 70.5%). Then, the LCA was performed on the remaining 395 patients using MPlus Version 8.4.^76^

Estimation was carried out with full information maximum likelihood with standard error and a χ^2^ test that are robust to non-normality and non-independence of observations (“estimator=MLR”). Model fit was evaluated to identify the solution that best characterized the observed latent class structure with the Bayesian Information Criterion (BIC), Vuong-Lo-Mendell-Rubin likelihood ratio test (VLMR), entropy, and latent class percentages that were large enough to be reliable.^77^ Missing data were accommodated for with the use of the Expectation-Maximization (EM) algorithm.^78^

Differences among the latent classes in demographic, clinical, and symptom characteristics, as well as stress and resilience measures, were evaluated using parametric and nonparametric tests. A value of *P* < .05 was considered statistically significant. Bonferroni corrected value of *P* < .008 was considered statistically significant for the pairwise contrasts.

## RESULTS

### Latent Class Solution

As noted in our previous publication,^26^ the 943 patients (70.5%) who had ≤1 occurrence of shortness of breath over the six assessments were classified as the None class. For the remaining 395 patients whose data were entered into the LCA, a three-class solution was selected because the BIC for that solution was lower than the BIC for the 2-class solution. In addition, the VLMR was significant for the 3-class solution, indicating that three classes fit the data better than two classes. Shortness of breath classes were labeled based on their distinct trajectories for the occurrence: Decreasing (8.2%), Increasing (7.8%), and High (13.5%) (Supplemental Figure 1).

### Demographic and Clinical Characteristics

As noted in our previous publication,^26^ significant differences were found among the latent classes for many of the demographic and clinical characteristics (Supplemental Table 1). In brief, compared to the None class, the High class was more likely to live alone, less likely to be employed, and more likely to report a previous or current history of smoking. In addition, they were more likely to have multiple cancer treatments, more likely to have lung metastasis, more likely to be receiving chemotherapy on 21- or 28-day cycles, and more likely to self-report a diagnosis of osteoarthritis, back pain, and rheumatoid arthritis. Compared to the None class, the Decreasing and High classes had lower KPS scores, a higher number of comorbidities, higher SCQ scores, and were more likely to self-report a diagnosis of depression. Compared to the None and Decreasing classes, the High class was more likely to be older and more likely to have lung cancer.

### Stress and Resilience Scores

Compared to the None class, the Decreasing and High classes reported higher PSS total, IES-R total, IES-R intrusion, IES-R hyper-arousal, and LSC-R affected sum scores. Compared to the None class, the High class reported higher IES-R avoidance subscale and LSC-R total scores. Compared to the None and Increasing classes, the High class reported higher LSC-R PTSD sum scores and lower CDRS scores (Table 1).

### Occurrence of SLEs

Compared to the None class, the High class reported higher occurrence rates for physical neglect, sexual harassment, being forced to touch and have sex before the age of 16, and being separated or divorced. Compared to the None class, the Increasing and High classes reported higher occurrence rates for being forced to touch and have sex at ≥ 16 years. Compared to the None class, the Decreasing class reported a higher occurrence rate for having parents separated or divorced (Table 2).

### Symptom Severity Scores

Compared to the None class, the other three classes reported higher levels of morning fatigue and sleep disturbance. Compared to the None class, the Decreasing and High classes reported higher levels of depressive symptoms, trait and state anxiety, cognitive dysfunction, and pain interference and decrements in morning energy. Compared to the None class, the Decreasing and High classes were more likely to have both cancer and non-cancer pain. Compared to the None class, the Increasing and High classes reported higher levels of evening fatigue. Compared to the None class, the High class reported a higher worst pain intensity score (Table 3).

## DISCUSSION

As part of an extension of our previous study of subgroups of patients with distinct shortness of breath profiles,^26^ this analysis is the first to describe associations between shortness of breath and three types of stress, as well as resilience in a sample of patients with heterogeneous types of cancer. Equally important, associations between shortness of breath and common symptoms were evaluated. The common and distinct risk factors for shortness of breath across the Decreasing, Increasing, and High classes compared to the None class are summarized in Table 4. Given the paucity of research on shortness of breath in oncology patients, our results are compared with findings from the general population and patients with cardio-pulmonary disease.

### Stress Measures

Compared to the None class, patients in both the Decreasing and High classes reported higher levels of global stress. Similarly, in previous studies of palliative care patients^32^ and adolescent patients with asthma,^79^ the severity of shortness of breath was positively correlated with global stress. One potential explanation for this relationship, that warrants additional investigation, is that both disruptions in the HPA axis and increases in systemic inflammation occur in oncology patients receiving chemotherapy that contribute to the occurrence of dyspnea.^32,80^

In terms of cancer-related stress, compared to the None class, both the Decreasing and High classes reported higher IES-R total, intrusion, and hyperarousal scores. Of note, in the Decreasing and High classes, 23.1% and 14.0% met the criteria for partial PTSD and 14.4% and 23.4% met the criteria for PTSD, respectively. In addition, fears of recurrence and declines in physical function in oncology patients may contribute to the severity of PTSD symptoms.^34^ These findings suggest that both cancer-related stress and relatively high levels of PTSD result in decreases in the threshold of dyspnea perception. This hypothesis is supported by a study that found that individuals who had PTSD following the World Trades Center disaster were twice as likely to experience shortness of breath than individuals without PTSD.^81^

While both the Decreasing and High classes showed similar trends in the severity of global and cancer-specific stress at enrollment, no explanation is readily apparent for why this pattern was not observed in the Increasing class. The distinct trajectories of shortness of breath for the Increasing class compared to the Decreasing class may be explained by differences in the provision of timely symptom management inventions (eg, steroids, oxygen, opioids, thoracentesis); different etiologies for shortness of breath (eg, anemia, pleural effusion); and/or the presence of a variety of triggers (eg, smoking, air pollution). If these trajectories are replicated in an independent sample, additional phenotypic characteristics and interventions warrant evaluation to determine the specific risk factors for membership in the Increasing class.

In terms of the overall number of SLEs, as well as the occurrence of specific SLEs, the primary differences were between the None and the High classes. Specifically, the High class reported higher occurrence rates for physical neglect, sexual harassment, forced to touch at <16 years, forced sex at <16 years of age, and being separated or divorced. In addition, compared to both the None and Increasing classes, the High class reported a higher LSC-R PTSD sum score. Our findings are consistent with previous reports that found that individuals who experienced interpersonal violence, abuse, and neglect had higher levels of PTSD symptoms, compared to those with other types of trauma (eg, unexpected death of a loved one).^82^

These findings suggest that for the High class in particular, the cumulative impact of various types of stress, particularly adverse childhood experiences (ACEs) may contribute to the occurrence of shortness of breath in patients receiving chemotherapy. Accumulating evidence suggests that exposure to early psychological stress contributes to the lifelong responsiveness of the HPA axis to stress.^83^ Specifically, repeated activation of the HPA axis may result in blunted cortisol responses to a variety of stressors (eg, airway inflammation).^31^ Over time, reduced inhibitory feedback associated with stress contributes to airway sensitization and chronic/refractory dyspnea.^31^ This hypothesis is supported by a study of the general population^84^ that found that exposure to a higher number of traumatic events and the occurrence of PTSD were associated with increased airflow limitations.^85^ Given the paucity of research on associations between dyspnea and SLEs in oncology patients, additional mechanistic studies are warranted.

### Co-occurring Symptoms

Anxiety is hypothesized to play a crucial role in dyspnea perception.^86^ Specifically, anxiety is known to amplify shortness of breath by increasing anxiety sensitivity (ie, the fear of anxiety symptoms^87^) and activating the limbic system.^88,89^ Therefore, it is not surprising that across our four distinct shortness of breath profiles, both trait and state anxiety scores exceeded the clinically meaningful cutoffs. Our findings are supported by a study of healthy volunteers,^90^ that found that higher trait anxiety was associated with dyspnea unpleasantness, as well as higher state anxiety levels. Additional investigations are warranted on the common and distinct roles of trait and state anxiety in the affective dimension of shortness of breath.

Depressive symptoms scores approached or exceeded cutoff scores only for the Decreasing and High classes. Evidence suggests that depression decreases the threshold of shortness of breath by altering the perception of respiratory sensations.^89^ Of note, in studies of patients with COPD,^91,92^ depression scores were positively correlated with the frequency of shortness of breath; functional impairment related to shortness of breath; emotional and cognitive responses to shortness of breath; and catastrophic thinking. In addition, in a study of patients with lung cancer that used latent variable modeling to create subgroups of patients with distinct functional status profiles,^37^ patients with the Severe Disability profile reported higher levels of shortness of breath and depressive symptoms. In terms of interventions for the co-occurrence of dyspnea and depression, in a study of patients with advanced cancer,^93^ the administration of sertraline resulted in decreases over time in the severity of both depression and shortness of breath. Additional research is warranted on the efficacy of antidepressants to decrease one or both of these symptoms.

Consistent with previous findings,^15,16,39,42,44^ morning fatigue scores exceeded clinically meaningful cutoffs in the Decreasing, Increasing, and High classes. This finding is not surprising, given that compared to the None class, these patients reported higher levels of sleep disturbance. In terms of evening fatigue, while all three classes of patients with shortness of breath had scores above the clinically meaningful cutoff, only the Decreasing and High classes had higher scores than the None class. Additional research is warranted to understand the specific causes of fatigue associated with shortness of breath in patients undergoing chemotherapy (eg, respiratory muscle weakness, hypoxia).

Consistent with four studies,^11,21,24,42^ patients in the High class reported higher worst pain scores. In addition, patients in both the Decreasing and High classes were more likely to have both cancer and noncancer pain. This finding is aligned with a longitudinal study of a large cohort of Medicare recipients that reported that compared to individuals without dyspnea, individuals with the symptom had higher pain prevalence rates (ie, 18% versus 64%).^94^ In addition, individuals with chest, back, or arthritis pain were substantially more likely to report dyspnea. Of note, the relative risk of dyspnea resolving was greatly increased if the pain problem resolved. One potential explanation for this association is that long-term physical inactivity associated with chronic pain results in physical deconditioning and secondary shortness of breath.^94^ Another potential explanation for the inter-connectedness between the two symptoms is that the perceptions of pain and shortness of breath activate similar brain cortical regions and shares common neural mechanisms.^95,96^

While patients in all four of our shortness of breath profiles had GSDS scores above the clinically meaningful cutoff, consistent with two studies of patients with advanced cancer,^15,39^ compared to the None class, the other three classes reported higher levels of sleep disturbance. Of note, normal human sleep causes a rapid and shallow breathing pattern; an unpredictable depth of breathing; a significant decrease in tidal volume; and decrements in ventilation and gas exchange during rapid eye movement (REM) sleep.^97^ Given the interconnectedness between sleep and respiration, shortness of breath during the night may contribute to decrements in sleep duration and sleep quality and increases in daytime tiredness (ie, morning fatigue). In terms of potential interventions, in a study of patients with COPD,^98^ progressive relaxation exercises improved dyspnea, fatigue, and sleep disturbance. The improvements in all three symptoms may be the result of increases in lung function and skeletal muscle relaxation, and/or decreases in stress.

Consistent with a previous finding in patients with COPD, asthma, or heart failure,^48^ higher occurrence rates of shortness of breath were associated with more severe decrements in cognitive function on our sample. In addition, in a study of healthy volunteers,^99^ experimentally induced dyspnea interfered with cognitive function. In other studies of community-dwelling older adults^100^ and patients with COPD,^101^ the co-occurrence of depression, anxiety, and sleep disturbance was associated with decrements in cognitive function.

### Resilience

All four of our classes had CDRS scores below the normative data for adults in the United States.^63^ Specifically, compared to both the None and Increasing classes, the High class had significantly lower resilience scores. Given that the High class reported a higher comorbidity burden, a lower functional status, a lower level of social support, and poorer quality of life,^26^ this finding is not surprising. Likewise, in patients with pulmonary disease,^102^ lower levels of resilience were correlated with higher levels of anxiety and depression and lower quality of life scores. Repetitive episodes of shortness of breath may cause fear that leads to the avoidance of daily activity or social interactions, neuropsychological symptoms, and decreases in resilience.^15,103,104^ However, because social buffering may modulate HPA reactivity to stressors,^105^ resilience training may decrease shortness of breath in oncology patients.^106^

### Limitations

Several limitations warrant consideration. Given that our sample was relatively homogeneous in terms of gender, race, and ethnicity, our findings may not generalize to more diverse racial and ethnic groups. Given that the major reason for refusal to participate was being overwhelmed with cancer and its treatments, stress levels in these patients may represent underestimates. While this study used a valid and reliable measure to assess the subjective experience of shortness of breath, future studies need to evaluate for correlations with objective measures of pulmonary function and/or neuroimaging. In addition, detailed information is needed on the etiology of shortness of breath and the use of pharmacologic and nonpharmaco-logic treatments. Finally, a more detailed evaluation of demographic and clinical characteristics associated with membership in the Increasing class warrants investigation.

## CONCLUSION

Despite these limitations, this study provides new information on the role of stress, resilience, and co-occurring symptoms in the occurrence of shortness of breath in a large sample of patients with heterogenous types of cancer. While some risk factors associated with shortness of breath in the Decreasing and High classes were similar, the High class reported higher rates of ACEs that may contribute to the higher rates of shortness of breath. While evidence-based interventions for dyspnea are lacking,^3^ clinicians can use the findings from this study to identify patients at increased risk for dyspnea during chemotherapy. In addition, given the high levels of stress and symptom burden in these patients, individualized symptom management interventions may decrease dyspnea. Equally important, patients may benefit from referrals to psychological services for stress management and cognitive-behavioral therapy. Additional mechanistic studies may increase our understanding of differences among the distinct shortness of breath profiles.

## Supplementary Material

2

1

## Figures and Tables

**TABLE 1:** Differences in Co-Occurring Symptom Severity Scores at Enrollment Among the Shortness of Breath Latent Classes.

Symptoms^a^	None (0) 70.5% (n = 934) Mean (SD)	Decreasing (1) 8.2% (n = 109) Mean (SD)	Increasing (2) 7.8% (n = 105) Mean (SD)	High (3) 13.5% (n = 181) Mean (SD)	Statistics
Depressive symptoms (≥16.0)	11.7 (9.1)	15.9 (11.0)	13.9 (10.3)	16.3 (10.4)	F = 16.47, *P* < .001 0 < 1 and 3
Trait anxiety (≥31.8)	34.1 (10.0)	38.0 (10.5)	35.1 (10.9)	38.8 (11.5)	F = 13.08, *P* < .001 0 < 1 and 3, 2 < 3
State anxiety (≥32.2)	32.9 (11.7)	36.5 (13.9)	33.6 (13.4)	37.6 (13.2)	F = 8.93, *P* < .001 0 < 1 and 3
Morning fatigue (≥3.2)	2.8 (2.2)	4.3 (2.1)	3.7 (2.3)	3.9 (2.3)	F = 26.70, *P* < .001 0 < 1, 2, and 3
Evening fatigue (≥5.6)	5.2 (2.2)	5.7 (2.0)	5.9 (1.9)	5.7 (1.9)	F = 6.82, *P* < .001 0 < 2 and 3
Morning energy (≤6.2)	4.6 (2.3)	4.0 (2.0)	4.1 (2.3)	3.9 (2.0)	F = 7.49, *P* < .001 0 > 1 and 3
Evening energy (≤3.5)	3.6 (2.0)	3.7 (2.0)	3.2 (2.2)	3.5 (2.0)	F=15.03, *P* < .001 0 > 1 and 3,1 < 2
Sleep disturbance (≥43.0)	50.0 (20.0)	58.5 (19.9)	55.8 (18.7)	59.8 (19.9)	F = 16.91, *P* < .001 0 < 1, 2, and 3
Attentional function (<5.0 = low, 5 to 7.5 = moderate, >7.5 = high)	6.6 (1.8)	5.6 (1.7)	6.4 (1.8)	5.9 (1.8)	F = 15.03, *P* < .001 0 > 1 and 3, 1 < 2
	% (n)	% (n)	% (n)	% (n)	
Type of pain					χ^2^ = 42.55, *P* <.001
No pain	30.5 (282)	17.9 (19)	27.9 (29)	16.4 (29)	0 > 1 and 3
Only noncancer pain	25.6 (237)	27.4 (29)	22.1 (23)	32.2 (57)	NS
Only cancer pain	17.7 (164)	11.3 (12)	12.5 (13)	11.3 (20)	NS
Both cancer and noncancer pain	26.2 (243)	43.4 (46)	37.5 (39)	40.1 (71)	0 < 1 and 3
For patients with pain	Mean (SD)	Mean (SD)	Mean (SD)	Mean (SD)	
Worst pain score	5.9 (2.5)	6.5 (2.5)	6.1 (2.9)	6.7 (2.4)	F = 4.55, *P* =.004 0<3
Mean pain interference score	2.7 (2.4)	3.7 (2.8)	3.3 (2.6)	4.2 (2.4)	F = 15.77, *P* < .001 0 < 1 and 3

Abbreviation: SD = standard deviation.

a Clinically meaningful cutoff scores.

**TABLE 2:** Differences in Stress and Resilience Measures Among the Shortness of Breath Latent Classes at Enrollment.

Measures^a^	None (0) 70.5% (n = 934) Mean (SD)	Decreasing (1) 8.2% (n = 109) Mean (SD)	Increasing (2) 7.8% (n = 105) Mean (SD)	High (3) 13.5% (n = 181) Mean (SD)	Statistics
PSS total score (range 0 to 56)	17.8 (8.0)	20.7 (8.2)	18.7 (8.3)	20.9 (8.1)	F = 10.19, *P* < .001 0 < 1 and 3
IES-R total score	17.5 (11.9)	21.7 (14.9)	19.9 (15.2)	23.2 (15.6)	F = 11.68, *P* < .001 0 < 1 and 3
(≥24.0 – clinically meaningful PTSD symptomatology)					
(≥33.0 – probable PTSD)					
IES-R intrusion	0.8 (0.6)	1.1 (0.8)	1.0 (0.8)	1.1 (0.8)	F = 10.46, *P* < .001 0 < 1 and 3
IES-R avoidance	0.9 (0.6)	1.0 (0.7)	1.0 (0.7)	1.1 (0.7)	F = 3.58, *P* = .013 0 < 3
IES-R hyperarousal	0.6 (0.6)	0.9 (0.8)	0.7 (0.8)	0.9 (0.8)	F = 15.14, *P* < .001 0 < 1 and 3
LSC-R total score (range 0–30)	5.7 (3.6)	6.7 (4.5)	6.5 (4.4)	7.4 (4.5)	F = 8.91, *P* < .001 0< 3
LSC-R affected sum score (range 0–150)	10.8 (9.5)	14.1 (13.8)	13.0 (12.3)	15.2 (13.0)	F = 8.56, *P* < .001 0 < 1 and 3
LSC-R PTSD sum score (range 0–21)	2.8 (2.7)	3.6 (3.7)	3.1 (3.1)	4.2 (3.7)	F = 8.94, *P* < .001 0 and 2 < 3
CDRS total score (range 0–40)	30.3 (6.4)	28.9 (6.3)	31.3 (5.7)	28.8 (6.4)	F = 5.23, *P* = .001 0 and 2 > 3, 1 < 2
(31.8 (±5.4) – normative mean score for the United States population)					

Abbreviations: CDRS = Connor Davidson Resilience Scale, IES-R = Impact of Event Scale – Revised, LSC-R = Life Stressor Checklist-Revised, PSS = Perceived Stress Scale, PTSD = post-traumatic stress disorder, SD = standard deviation.

a Clinically meaningful cutoff scores or range of scores.

**TABLE 3:** Differences Among the Shortness of Breath Latent Classes in the Percentage of Patients Exposed to Specific Stressors.

Stressful Life Event	None (0) 70.5% (n = 943) % (n)	Decreasing (1) 8.2% (n = 109) % (n)	Increasing (2) 7.8% (n = 105) % (n)	High (3) 13.5% (n = 181) % (n)	Statistics
Interpersonal Violence, Abuse, and Neglect Stressors					
Family violence in childhood	22.7 (161)	26.4 (23)	17.8 (16)	31.2 (43)	χ^2^ = 6.73, *P* = .081
Emotional abuse	19.4 (138)	25.6 (23)	24.2 (22)	29.1 (41)	χ^2^ = 7.83, *P* = .050
Physical neglect	3.7 (26)	8.8 (8)	3.3 (3)	9.3 (13)	χ^2^ = 11.69, *P* = .009 0 < 3
Sexual harassment	15.1 (106)	22.5 (20)	24.2 (22)	27.1 (38)	χ^2^ = 15.41, *P* = .001 0 < 3
Physical abuse - <16 years	12.3 (87)	21.3 (19)	16.5 (15)	18.4 (26)	χ^2^ = 8.18, *P* = .042 no significant pairwise contrasts
Physical abuse - ≥16 years	12.2 (86)	19.3 (17)	14.3 (13)	15.7 (22)	χ^2^ = 4.22, *P* = .239
Forced to touch - <16 years	9.2 (65)	13.5 (12)	15.7 (14)	19.9 (28)	χ^2^ = 14.91, *P* = .002 0 < 3
Forced to touch - ≥16 years	4.4 (31)	7.9 (7)	11.1 (10)	9.9 (14)	χ^2^ = 11.61, *P* = .009 0 < 2 and 3
Forced sex - <16 years	2.8 (20)	7.8 (7)	5.6 (5)	9.2 (13)	χ^2^ = 14.53, *P* = .002 0 < 3
Forced sex - ≥16 years	4.8 (34)	6.7 (6)	12.2 (11)	10.6 (15)	χ^2^ = 12.16, *P* = .007 0 < 2 and 3
Other Stressors					
Been in a serious disaster	40.3 (286)	41.1 (37)	42.9 (39)	42.4 (59)	χ^2^ = 0.40, *P* = .941
Seen serious accident	31.9 (227)	26.7 (24)	31.9 (29)	41.1 (58)	χ^2^ = 6.27, *P* = .099
Had serious accident or injury	23.2 (163)	23.3 (21)	25.6 (23)	29.3 (41)	χ^2^ = 2.49, *P* = .477
Jail (family member)	18.3 (130)	26.7 (24)	26.7 (24)	24.5 (34)	χ^2^ = 7.65, *P* = .054
Jail (self)	6.3 (45)	8.9 (8)	6.7 (6)	7.8 (11)	χ^2^ = 1.12, *P* = .773
Foster care or put up for adoption	2.2 (16)	2.2 (2)	3.3 (3)	2.8 (4)	χ^2^ = 0.51, *P* = .917
Separated/divorced (parents)	19.2 (137)	31.5 (28)	25.3 (23)	26.2 (37)	χ^2^ = 10.02, *P* = .018 0 < 1
Separated/divorced (self)	33.4 (239)	41.1 (37)	35.6 (32)	47.1 (66)	χ^2^ = 10.69, P =.014 0 < 3
Serious money problems	18.2 (130)	21.1 (19)	24.7 (22)	24.8 (35)	χ^2^ = 4.80, *P* = .187
Had serious physical or mental illness (not cancer)	16.9 (121)	20.9 (19)	22.0 (20)	26.1 (37)	χ^2^ = 7.34, *P* = .062
Abortion or miscarriage	43.8 (236)	46.9 (38)	41.3 (31)	46.2 (55)	χ^2^ = 0.73, *P* = .867
Separated from child	1.6 (11)	2.3 (2)	3.4 (3)	3.7 (5)	χ^2^ = 3.21, *P* = .360
Care for child with handicap	3.8 (26)	4.6 (4)	3.4 (3)	4.4 (6)	χ^2^ = 0.31, *P* = .958
Care for someone with severe physical or mental handicap	22.9 (161)	23.9 (21)	23.9 (21)	32.6 (45)	χ^2^ = 5.88, *P* = .117
Death of someone close (sudden)	49.0 (344)	51.7 (46)	50.5 (46)	48.9 (67)	χ^2^ = 0.29, *P* = .962
Death of someone close (not sudden)	78.9 (551)	76.7 (66)	79.5 (70)	80.6 (112)	χ^2^ = 0.49, *P* = .921
Seen robbery/mugging	21.2 (151)	23.6 (21)	18.7 (17)	27.3 (38)	χ^2^ = 3.28, *P* = .350
Been robbed/mugged	25.6 (181)	30.3 (27)	30.8 (28)	26.6 (37)	χ^2^ = 1.78, *P* = .619

**TABLE 4:** Characteristics Associated with Membership in the Decreasing, Increasing, and High Shortness of Breath Classes.

Characteristic^a^	Decreasing	Increasing	High
Symptom Characteristics			
Higher depressive symptoms	■		■
Higher trait anxiety	■		■
Higher state anxiety	■		■
Higher morning fatigue	■	■	■
Higher evening fatigue		■	■
Lower morning energy	■		■
Higher sleep disturbance	■	■	■
Lower attentional function	■		■
Less likely not to have pain	■		■
More likely to have both cancer and non-cancer pain	■		■
Higher worst pain score			■
Higher mean pain interference score	■		■
Stress and Resilience Measures			
Higher Perceived Stress Scale score	■		■
Higher Impact of Event Scale-Revised total score	■		■
Higher Impact of Event Scale-Revised intrusion score	■		■
Higher Impact of Event Scale-Revised avoidance score			■
Higher Impact of Event Scale-Revised hyperarousal score	■		■
Higher Life Stressor Checklist-Revised total score			■
Higher Life Stressor Checklist-Revised affected sum score	■		■
Higher Life Stressor Checklist-Revised PTSD sum score			■
Lower Connor Davidson Resilience Scale total score			■
Higher Occurrence of Interpersonal Violence, Abuse, and Neglect Stressors			
Physical neglect			■
Sexual harassment			■
Forced to touch <16 years			■
Forced to touch ≥16 years		■	■
Forced sex <16 years			■
Forced sex ≥16 years		■	■
Higher Occurrence of Other Stressors			
Separated/divorced (parents)	■		
Separated/divorced (self)			■

■ Indicates that the class had this characteristic compared to the None class.

Abbreviation: PTSD = post-traumatic stress disorder.

a Comparisons done with the None group.
