# Supplementary material for: Higher Lifetime Stress and Symptom Burden Contribute to the Occurrence of Shortness of Breath
Source: Semin Oncol Nurs. Author manuscript; Available in PMC 2024 Oct 2. (PMC11446157; doi:10.1016/j.soncn.2023.151471)
Supplement: 2 [file NIHMS2025528-supplement-2.pdf]

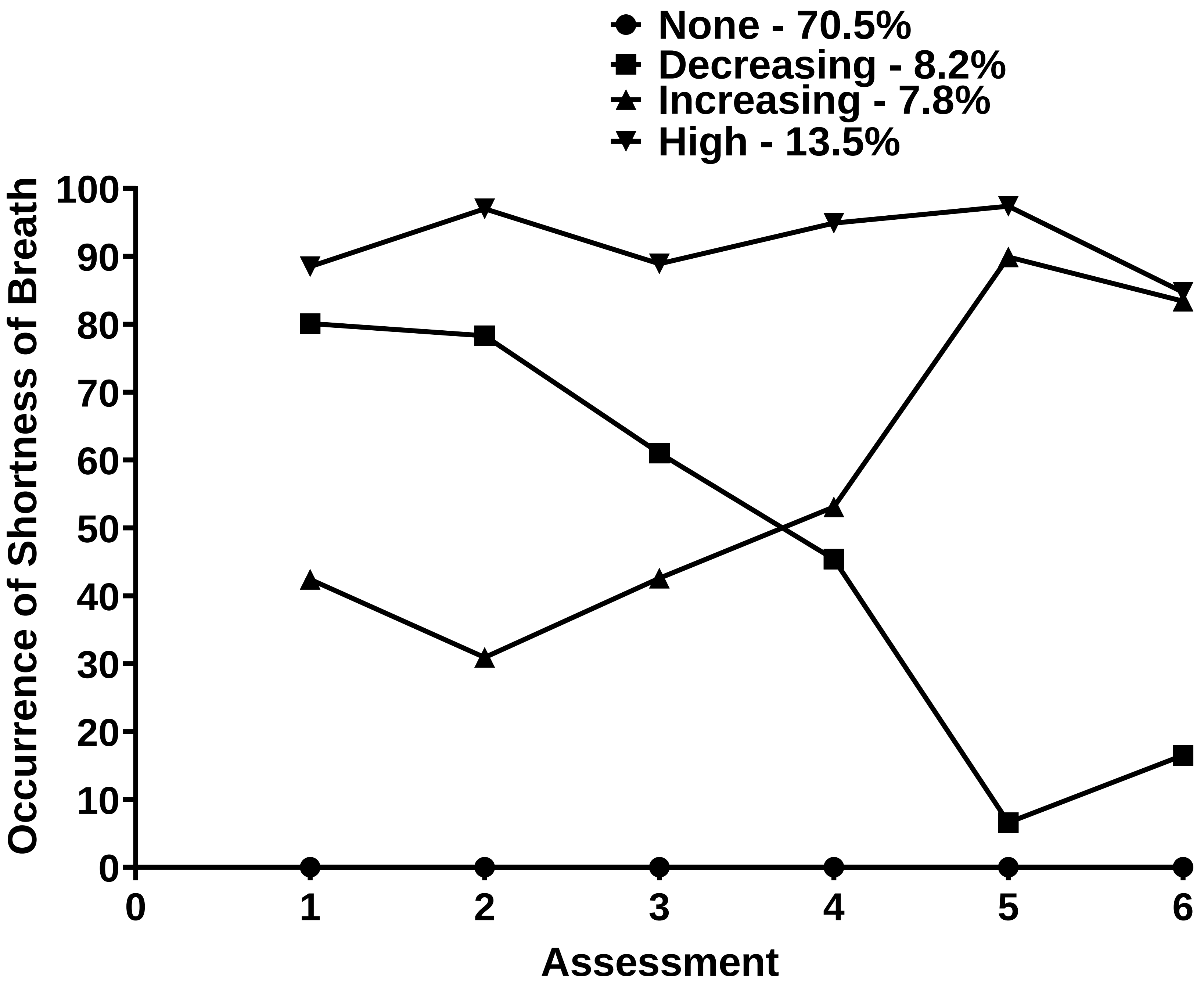

Supplemental Figure 1 - Subgroups of patients with distinct shortness of breath profiles who were assessed over two cycles of chemotherapy (i.e., prior to the next dose of chemotherapy (assessments 1 and 4), approximately one week after the receipt of' chemotherapy (assessments 2 and 5) and approximately two weeks after the receipt of chemotherapy (i.e., assessments 3 and 6).
