# Supplementary material for: Higher Lifetime Stress and Symptom Burden Contribute to the Occurrence of Shortness of Breath
Source: Semin Oncol Nurs. Author manuscript; Available in PMC 2024 Oct 2. (PMC11446157; doi:10.1016/j.soncn.2023.151471)
Supplement: 1 [file NIHMS2025528-supplement-1.docx]

Supplementary Table 1 – Differences in Demographic and Clinical Characteristics at Enrollment Among the Shortness of Breath Latent Classes at Enrollment

| Characteristic | None (0)  70.5% (n=943) | Decreasing (1)  8.2% (n=109) | Increasing (2)  7.8% (n=105) | High (3)  13.5% (n=181) | Statistics |
| --- | --- | --- | --- | --- | --- |
|  | Mean (SD) | Mean (SD) | Mean (SD) | Mean (SD) |  |
| Age (years) | 57.0 (12.3) | 55.3 (13.0) | 56.5 (12.2) | 59.6 (12.0) | F = 3.44, p = .016  0 and 1 < 3 |
| Education (years) | 16.3 (3.0) | 15.8 (2.8) | 16.1 (3.0) | 16.2 (3.1) | F = 0.69, p = .558 |
| Body mass index (kilogram/meter squared) | 25.9 (5.4) | 26.2 (6.4) | 27.4 (6.5) | 26.8 (6.0) | F = 3.05, p = .028  no significant pairwise contrasts |
| Alcohol Use Disorders Identification Test score | 3.1 (2.5) | 2.9 (2.3) | 2.5 (2.5) | 2.8 (2.5) | F = 1.11, p = .346 |
| Karnofsky Performance Status score | 81.7 (12.0) | 75.4 (12.4) | 78.9 (12.9) | 74.7 (12.7) | F = 21.31, p <.001  0 > 1 and 3, 2 > 3 |
| Number of comorbid conditions | 2.2 (1.3) | 2.7 (1.6) | 2.5 (1.5) | 3.1 (1.6) | F = 21.05, p <.001  0 < 1 and 3, 2 < 3 |
| Self-administered Comorbidity Questionnaire score | 5.1 (2.8) | 6.2 (3.5) | 5.8 (3.6) | 7.1 (4.0) | F = 24.27, p <.001  0 < 1 and 3, 2 < 3 |
| Time since diagnosis (years) | 1.7 (3.2) | 2.7 (5.5) | 2.3 (4.0) | 2.8 (5.3) | KW = 10.42, p = .015  no significant pairwise contrasts |
| Time since diagnosis (years, median) | 0.41 | 0.50 | 0.44 | 0.51 |  |
| Number of prior cancer treatments | 1.5 (1.4) | 1.9 (1.7) | 1.7 (1.5) | 1.8 (1.7) | F = 4.27, p = .005  0 < 3 |
| Number of metastatic sites including lymph node involvement^a^ | 1.2 (1.2) | 1.2 (1.2) | 1.3 (1.2) | 1.4 (1.4) | F = 1.06, p = .365 |
| Number of metastatic sites excluding lymph node involvement | 0.8 (1.0) | 0.8 (1.0) | 0.8 (1.1) | 1.0 (1.2) | F = 1.84, p = .138 |
| MAX2 score | 0.17 (0.08) | 0.17 (0.08) | 0.18 (0.08) | 0.18 (0.09) | F = 0.60, p = .613 |
|  | % (n) | % (n) | % (n) | % (n) |  |
| Gender (% female) | 75.2 (708) | 89.0 (97) | 84.8 (89) | 80.7 (146) | Χ^2^ = 15.51, p = .001  0 < 1 |
| Self-reported ethnicity  White  Asian or Pacific Islander  Black  Hispanic, Mixed, or Other | 69.6 (649)  13.2 (123)  6.6 (62)  10.6 (99) | 61.7 (66)  14.0 (15)  12.1 (13)  12.1 (13) | 67.0 (69)  13.6 (14)  7.8 (8)  11.7 (12) | 76.4 (136)  7.3 (13)  6.7 (12)  9.6 (17) | Χ^2^ = 11.41, p = .249 |
| Married or partnered (% yes) | 67.0 (623) | 62.6 (67) | 52.9 (54) | 58.9 (106) | Χ^2^ = 11.10, p = .011  0 > 2 |
| Lives alone (% yes) | 19.7 (183) | 17.6 (19) | 30.1 (31) | 28.5 (51) | Χ^2^ = 12.50, p = .006  0 < 3 |
| Currently employed (% yes) | 37.3 (348) | 30.6 (33) | 34.6 (36) | 26.7 (48) | Χ^2^ = 8.66, p = .034  0 > 3 |
| Annual household income  Less than $30,000^+^  $30,000 to $70,000  $70,000 to $100,000  Greater than $100,000 | 14.8 (125)  21.2 (179)  17.4 (147)  46.6 (394) | 28.9 (28)  18.6 (18)  14.4 (14)  38.1 (37) | 26.4 (24)  24.2 (22)  13.2 (12)  36.3 (33) | 26.1 (43)  20.0 (33)  18.2 (30)  35.8 (59) | KW = 19.53, p <.001  0 > 2 and 3 |
| Child care responsibilities (% yes) | 22.8 (211) | 22.9 (24) | 22.8 (23) | 18.2 (32) | Χ^2^ = 1.86, p = .602 |
| Elder care responsibilities (% yes) | 7.4 (64) | 7.4 (7) | 14.1 (14) | 7.0 (11) | Χ^2^ = 5.78, p = .123 |
| Past or current history of smoking (% yes) | 32.9 (306) | 37.0 (40) | 37.6 (38) | 45.5 (81) | Χ^2^ = 10.76, p = .013  0 < 3 |
| Level of exercise  Does not exercise on a regular basis  Exercises less than 150 minutes per week  Exercises 150 or more minutes per week | 35.1 (251)  45.1 (323)  19.8 (142) | 40.9 (36)  35.2 (31)  23.9 (21) | 39.2 (31)  44.3 (35)  16.5 (13) | 43.5 (64)  41.5 (61)  15.0 (22) | Χ^2^ = 7.66, p = .264 |
| Specific comorbid conditions (% yes)  Heart disease  High blood pressure  Lung disease  Diabetes  Ulcer or stomach disease  Kidney disease  Liver disease  Anemia or blood disease    Depression    Osteoarthritis    Back pain    Rheumatoid arthritis | 5.1 (48)  30.1 (284)  7.5 (71)  8.2 (77)  4.5 (42)  1.1 (10)  6.0 (57)  10.3 (97)  15.4 (145)  10.4 (98)  22.2 (209)  2.9 (27) | 6.4 (7)  32.1 (35)  14.7 (16)  15.6 (17)  5.5 (6)  1.8 (2)  5.5 (6)  19.3 (21)  27.5 (30)  11.9 (13)  33.0 (36)  1.8 (2) | 3.8 (4)  25.7 (27)  10.5 (11)  7.6 (8)  4.8 (5)  1.0 (1)  9.5 (10)  18.1 (19)  22.9 (24)  16.2 (17)  31.4 (33)  1.0 (1) | 9.9 (18)  32.6 (59)  29.3 (53)  10.5 (19)  6.6 (12)  3.3 (6)  7.2 (13)  14.9 (27)  32.0 (58)  19.3 (35)  36.5 (66)  7.2 (13) | Χ^2^ = 7.45, p = .059  Χ^2^ = 1.68, p = .641  Χ^2^ = 73.16, p <.001  0, 1, and 2 < 3  Χ^2^ = 7.30, p= .063  Χ^2^= 1.66, p = .645  Χ^2^ = 5.81, p = .121  Χ^2^ = 2.23, p = .526  Χ^2^ = 12.90, p= .005  0 < 1  Χ^2^ = 33.90, p <.001  0 < 1 and 3  Χ^2^ = 13.07, p = .004  0 < 3  Χ^2^ = 22.02, p <.001  0 < 3  Χ^2^ = 11.93, p = .008  0 < 3 |
| Cancer diagnosis  Breast cancer  Gastrointestinal cancer  Gynecological cancer  Lung cancer | 38.4 (362)  35.4 (334)  17.7 (167)  8.5 (80) | 51.4 (56)  21.1 (23)  15.6 (17)  11.9 (13) | 46.7 (49)  21.0 (22)  18.1 (19)  14.3 (15) | 39.8 (72)  16.6 (30)  16.6 (30)  27.1 (49) | Χ^2^ = 76.19, p <.001  NS  0 > 1, 2, and 3  NS  0 and 1 < 3 |
| Co-occurrence of lung cancer and lung disease | 56.3 (45) | 61.5 (8) | 40.0 (6) | 79.6 (39) | Χ^2^ = 10.68, p = .014  0 and 2 < 3 |
| Prior cancer treatment  No prior treatment  Only surgery, CTX, or RT  Surgery and CTX, or surgery and RT, or CTX and RT  Surgery and CTX and RT | 25.6 (235)  43.6 (400)  20.2 (185)  10.6 (97) | 19.8 (21)  41.5 (44)  20.8 (22)  17.9 (19) | 18.8 (19)  46.5 (47)  17.8 (18)  16.8 (17) | 28.2 (50)  31.6 (56)  18.6 (33)  21.5 (38) | Χ^2^ = 26.42, p = .002  NS  0 > 3  NS  0 < 3 |
| Receipt of targeted therapy (% yes) | 27.2 (251) | 33.3 (36) | 31.7 (33) | 40.9 (72) | Χ^2^ = 14.18, p = .003  0 < 3 |
| Cycle length  14 day cycle^+^  21 day cycle  28 day cycle | 45.5 (425)  48.0 (449)  6.5 (61) | 40.7 (44)  52.8 (57)  6.5 (7) | 41.0 (43)  49.5 (52)  9.5 (10) | 25.8 (46)  63.5 (113)  10.7 (19) | KW = 24.14, p <.001  0 < 3 |
| Metastatic sites  No metastasis  Only lymph node metastasis  Only metastatic disease in other sites  Metastatic disease in lymph nodes and other sites | 32.2 (299)  23.2 (216)  21.1 (196)  23.5 (219) | 34.0 (36)  17.9 (19)  20.8 (22)  27.4 (29) | 30.8 (32)  26.0 (27)  19.2 (20)  24.0 (25) | 33.3 (60)  16.1 (29)  22.8 (41)  27.8 (50) | Χ^2^ = 7.15, p = .622 |
| Lung metastasis (% yes) | 14.1 (89) | 21.1 (15) | 15.1 (11) | 30.0 (36) | Χ^2^ = 19.28, p <.001  0 < 3 |
| Emetogenicity of the CTX regimen  Minimal/low  Moderate  High | 18.1 (169)  63.2 (591)  18.7 (175) | 24.8 (27)  50.5 (55)  24.8 (27) | 19.0 (20)  56.2 (59)  24.8 (26) | 24.2 (43)  59.0 (105)  16.9 (30) | KW = 3.41, p = .332 |
| Antiemetic regimen  None  Steroid alone or serotonin receptor antagonist alone  Serotonin receptor antagonist and steroid  NK-1 receptor antagonist and two other antiemetics | 7.2 (66)  19.5 (178)  49.0 (448)  24.3 (222) | 7.6 (8)  22.9 (24)  41.0 (43)  28.6 (30) | 5.9 (6)  23.5 (24)  46.1 (47)  24.5 (25) | 6.9 (12)  22.3 (39)  45.7 (80)  25.1 (44) | Χ^2^ = 4.02, p = .910 |

^a^Total number of metastatic sites evaluated was 9.

^+^Reference group

Abbreviations: CTX = chemotherapy, KW = Kruskal Wallis, NK-1 = neurokinin-1, NS = not significant, RT = radiation therapy, SD = standard deviation
